# Moving model analysis on the transient pressure and slipstream caused by a metro train passing through a tunnel

**DOI:** 10.1371/journal.pone.0222151

**Published:** 2019-09-10

**Authors:** Shuang Meng, Dan Zhou, Zhe Wang

**Affiliations:** 1 Key Laboratory of Traffic Safety on Track (Central South University), Ministry of Education, Changsha, Hunan, China; 2 Joint International Research Laboratory of Key Technology for Rail Traffic Safety, Changsha, Hunan, China; 3 National & Local Joint Engineering Research Center of Safety Technology for Rail Vehicle, Changsha, Hunan, China; Southwest Jiaotong University, CHINA

## Abstract

In this study, the spatial distribution of the transient pressure and the slipstream caused by a 1/10 scaled metro train passing through a tunnel was studied with moving model test. We hereby investigate the mechanism underlying the mitigation of the transient pressure on both the train surface and tunnel wall, as well as that of the slipstream in the tunnel. Experimental results showed that the airshaft at different locations in a tunnel had different pressure relief effects. The most significant pressure amplitude decreased by 36.0% with the airshaft locating in the middle of the tunnel. Meanwhile, the slipstream speed was also relieved from 0.45 to 0.36 after an airshaft. We also assessed and analyzed the impact of train speed on the transient pressures and slipstream. It was found that the increase of the train speed would increase the transient pressure and slipstream speed, but it did not effect their spatial distribution.

## Introduction

As quick and efficient means of short-distance transportation, metro tunnels have been developed in many countries. Since the designed running speeds of existing metro-tunnel systems are generally low, the aerodynamic effect generated when a train travels through a tunnel is small. However, the speed of metro trains is increasing. For instance, the maximum speed of the metro train in San Francisco, U.S.A., is 128 km/h, and the maximum speed of Metro Line 3 in Guangzhou, China, is 120 km/h. Since metro tunnels suffer large blockage ratio, the trains induce strong transient pressures and slipstreams. These cause adverse impacts on the stability and safety of the train body and the tunnel structure, when a train is traveling in a tunnel. The characteristics of the strong transient pressure and the slipstream caused by a train passing through a tunnel, as well as the necessary mitigation measures, have already attracted extensive attention of scientific researchers.

Some scholars have applied numerical simulations to study the aerodynamic characteristics of a train traveling in a tunnel. Tunnel space is relatively closed, leading to large pressure variation amplitude and strong airflow when a train enters a tunnel [1–4]. For this purpose, some researchers have studied the impacts of different factors, such as the tunnel length, shape of the tunnel cross section, blockage ratio, shape of the train head, and train speed on the pressure waves and the train-induced unsteady flow in tunnels [5–8]. Niu et al. [9] researched the effect of train length on fluctuating aerodynamic pressure wave in tunnels and put up a method for determining the amplitude of pressure wave on trains. The results show that, because of the significant difference in pressure amplitude, long trains cannot be replaced by short trains when simulating trains going through or passing each other in tunnels. Yuan et al. [10] studied the unsteady aerodynamic performance of an inter-city train passing through a station in a tunnel, finding the asymmetrical internal structure of the station and the abrupt change in cross-section are the main factors contributing to the poor running stability of the inter-city train, with car 1 and car 8 showing the worst running stability. Zhang et al. [11–12] studied the radiation of train-tunnel impulse waves. Several tunnel exit geometries with and without a flange at the exit portal of the tunnel were simulated to check their effects on the radiation of impulse waves. The results show that the maximum magnitude of the impulse wave increases with the flange length at the far field. To relieve the strong transient pressure and the micro-pressure wave caused by a train traveling in a tunnel, some articles set up hoods at the tunnel opening and studied the relief effects of differently shaped of hoods on the pressure [13–15]. The results show that hoods at the opening can effectively reduce the pressure gradient in a tunnel and reduce the micro-pressure wave amplitude at the tunnel exit.

To improve air conditions and reduce pressures at a metro station, an airshaft is typically added near the station [16]. Some researchers have studied the impacts of adding airshafts on the aerodynamic effects in tunnels [17–19]. Xue et al. [20] analyzed the unsteady flow at a metro station and in a tunnel and found that the position of an airshaft had a significant impact on the tunnel airflow. González et al. [21] applied CFD to establish a three-dimensional numerical model for a metro station and a tunnel between stations. They then applied a dynamic mesh to simulate the unsteady flow in a tunnel and simultaneously analyze the impacts of the vertical shafts on the tunnel ventilation system. However, because numerical simulations are limited by mathematical models, geometrical models, and other relevant factors, they cannot completely reconstruct the real flow field, which needs to be further studied and tested.

Moving model test is a test of dynamics that simulates the movement of a train as it affects flow around it. This type of test is able to truly simulate the relative motion between a train and its ambient environment and accurately reflect the ground effect [22–23]. When studying the aerodynamic effect of a train passing through a tunnel, many researchers worldwide have employed a moving model test. For example, Kim et al. [24] applied a train model at a scale of 1/20 to analyze train-induced unsteady flow in a tunnel with a maximum test speed of 3 m/s and to obtain trends for the pressure and slipstream changes with time. With increasing train speed, the train-induced transient pressure and flow in a tunnel will be more pronounced. Doi et al. [25] studied the impacts of the shape of train heads on the gradient of the pressure wave in a tunnel, with a 1/30 scale train model. Additionally, Baker et al. [26] used a 1/25 scale train model to analyze the transient pressure on the surrounding structures when trains with three different head shapes were passing through. The results showed that the transient pressure peak was closely correlated to the type and shape of the ancillary facilities; when the train was more streamlined, the transient pressure was smaller. A simplified ICE2 1/25 scale train model was created to analyze the slipstream, finding that the slipstream speed reached a maximum near the train tail. Additionally, the impact of a freight train carrying different quantities of goods on a slipstream was analyzed, showing that the freight train slipstream speed was significantly larger than the slipstream speed of a passenger train. In addition, the impacts of the train length and speed on a slipstream were analyzed [27–28].

To study the impact of a changing tunnel structure on the interior train-induced aerodynamic effects, Miyachi et al. [29] created a 1/137 scale model to study the pressure waves generated when a train passed through a tunnel having branches. The results showed that cross-sectional area ratio of the branches was a key factor determining the pressure wave amplitude and radiation pulse at the tunnel entrance. Liu et al. [30] used a 1/31 scale model to study the aerodynamic effects in lined and unlined tunnels. Zhou et al. [31] employed a 1/20 scale train model to study the piston effect caused by the train passing through a platform. They also discussed the pressure characteristics on the train surface when the train was passing by the station and when two trains intersected at the station. Zhang et al. [32] used a 1/20 scale model to analyze the impact of differently shaped hood structures at a tunnel opening on the initial compression waves, showing that a hat oblique tunnel portal combined with a buffer structure with top holes was particularly effective. Gilbert et al. [33] made a 1/25 scale train model to study the slipstream under different blockage ratios and different tunnel lengths.

The above studies demonstrate that the moving model test is the most effective method that can presently be applied to the study of a metro train passing through a tunnel. However, limited by the model test bed and test conditions, the scale of the train models could not exceed 1/20. Niu et al. [34] studied the impacts of the Reynolds number on the aerodynamic pressure when the train was passing through tunnel, showing that the train model scale (Reynolds number) impacts the pressure amplitude. With increasing model scale, the pressure wave amplitude also increases and more closely resembles the results of a full-scale model. Additionally, most of the studies on the airshaft and the slipstream in a tunnel adopted numerical simulations, while few adopted the moving model test.

In this study, a 1/10 scaled train model was used to examine the aerodynamic pressure and slipstream generated while passing through a tunnel. To simulate the actual speed of a metro train, we tried to apply a higher test speed. When the metro train passed by a tunnel at 120 km/h, the pressure at the train surface and the tunnel wall, as well as the distribution characteristics of the slipstream were studied.

## Methodology

### Dynamic model unit and test model

**Fig 1:**
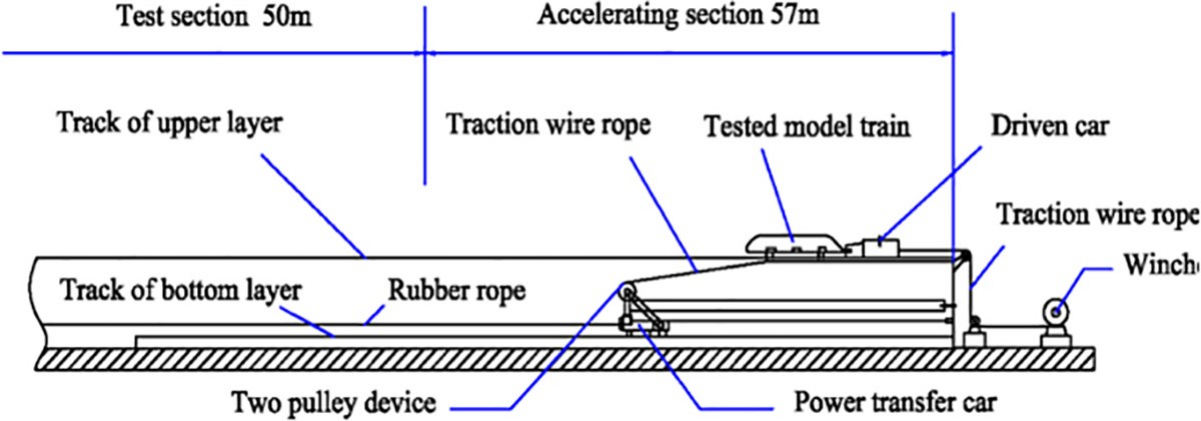
Schematic diagram of the moving model test platform.

The transient aerodynamic performance of a moving train can be tested by building a moving model test device [22–23,35]. The moving model test in this study was made at the Key Laboratory of Traffic Safety on Track, Central South University, China. The test line of the device was 164 m total length divided into three sections—acceleration, test, braking. The test section was 50 m. The accelerating system used accelerated the model train from zero to 500 km/h; then a step-by-step deceleration system decelerated it from 500 km/h to 0 in 0.5 s. To ensure that the pressure wave amplitude did not decrease, the rigid tunnel model was well sealed on the moving model test bed, allowing us to measure the transient pressure on the train surface, the transient pressure on the tunnel wall, and the slipstream in the tunnel. Before this, a series of tests, including a measurement of the pressure on the train surface, the pressure on the tunnel wall, and the pressure of the accessory device at the rail side, were performed on the moving model platform, and some reliable test results were acquired [30–32]. Fig 1 shows the schematic diagram of the moving model test platform.

**Fig 2:**
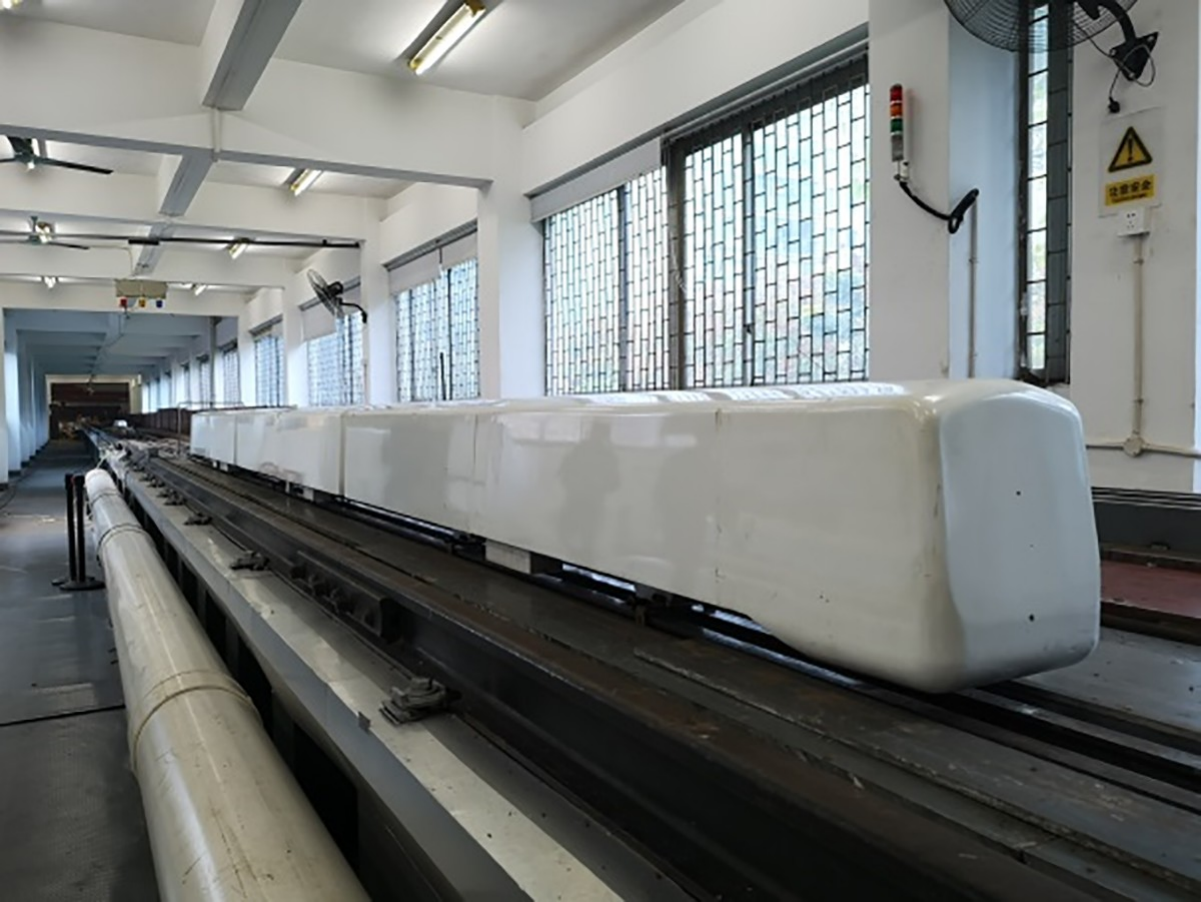
1/10-scale train model.

**Fig 3:**
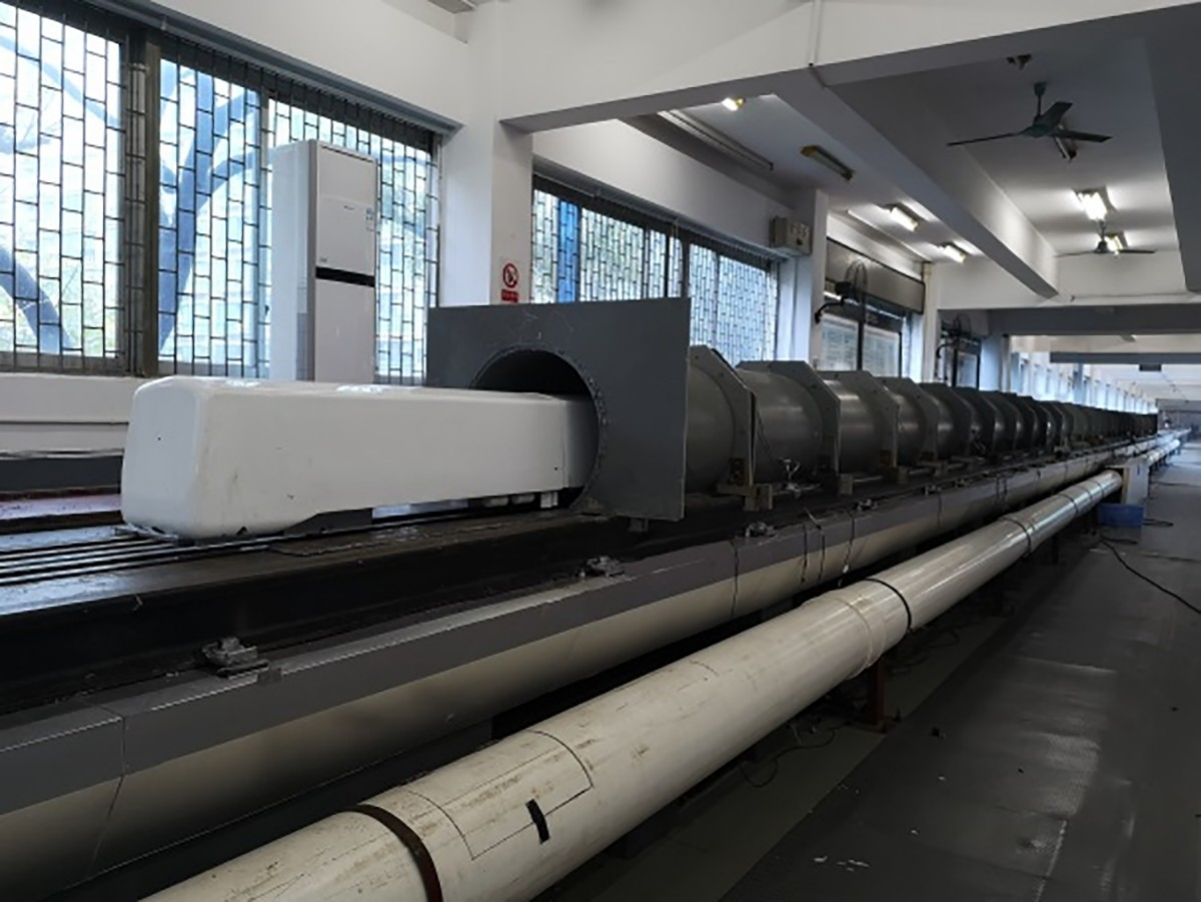
Train-tunnel model.

A 1/10-scale metro train model was used in this test. A scaled model of the test train should accurately represent the train head and tail. The train model consisted of a three-car multiple unit, including a head car, middle car, and tail car. To make the test results close to the actual operating conditions of the train, the parts that impacted aerodynamic performance, such as the windshield, bogie, and bottom hanger, were also simulated. To obtain an accurate, good quality model, the train model was 3D printed. The train model was 7.09 m long (L_tr_) ×0.313 m wide (W) ×0.367 m high (H), with a cross sectional area of 0.095 m^2^ and a windshield clearance of 0.07 m, as shown in Fig 2. The tunnel was a single-track tunnel with a cross sectional area of 0.22 m^2^. The ratio of the cross-sectional area of the train to the cross-sectional area of tunnel is the blockage ratio β. In this instance, β = 0.43. During the testing period of without airshaft, every airshaft was closed [36–37]. The train-tunnel model is shown in Fig 3.

### Layout of the coordinate system and the measuring points

**Fig 4:**
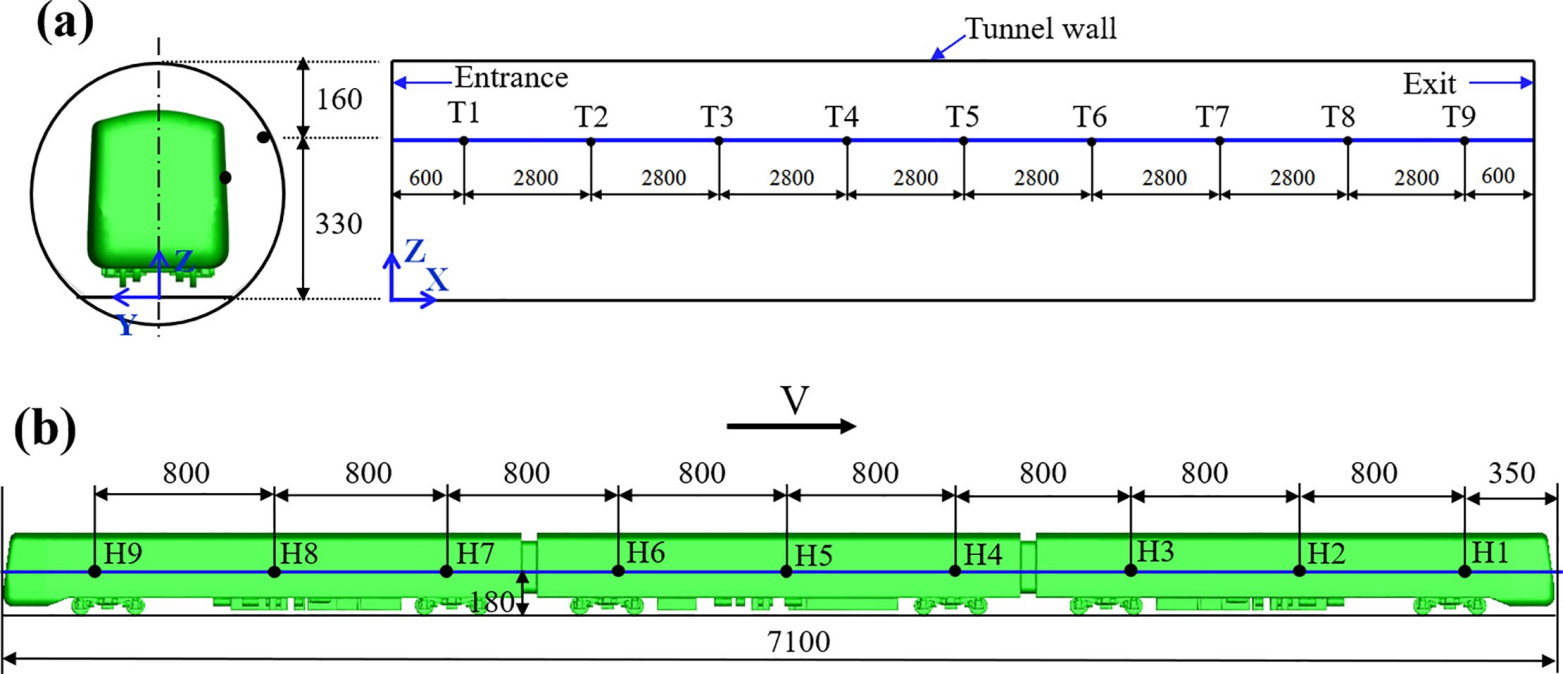
Layout of measurement points. (a) Measuring points in the tunnel, (b) Measuring points on the train body (unit: mm).

The coordinate system was defined as shown in Fig 4(A). The X-axis was defined as the direction of train travel, and the origin was defined as the central line of the tracks at the tunnel entrance. The Y-axis was defined in the horizontal plane perpendicular to the tracks and the Z-axis was perpendicular to the plane of tracks (vertical). Note that for a clearer visualization of the layout of measurement points and domain dimensions, Fig 4 is not drawn to scale. The pressure measuring points and the velocity measuring points in the tunnel were in the same position, as shown in Fig 4(A). The measuring points were 330 mm from the ground (*z*/*H* = 0.9), 20 mm from the tunnel wall. The first measuring point is 600 mm from the tunnel entrance, and the spacing between each measuring point is 2800 mm. The layout of the measuring points on the train surface is shown in Fig 4(B). Each point on the train body placed at *z*/*H* = 0.49 at equally-spaced intervals. Because both the train and the tunnel were symmetrical about the central line of the tracks, the measurement points on the train surface and tunnel wall were set on the same side.

### Data collection and processing

The data collection system consists of two parts: the train-mounted collection system and the ground collection system. The two collection systems were mutually independent but coordinated with synchronous control signals. The train-mounted collection system used to measure the pressure at the train surface was installed inside the train. The ground collection system was used to measure the speed of the model train at the entrance of the test section, the pressure in the tunnel, and the slipstream in the tunnel in real time.

**Fig 5:**
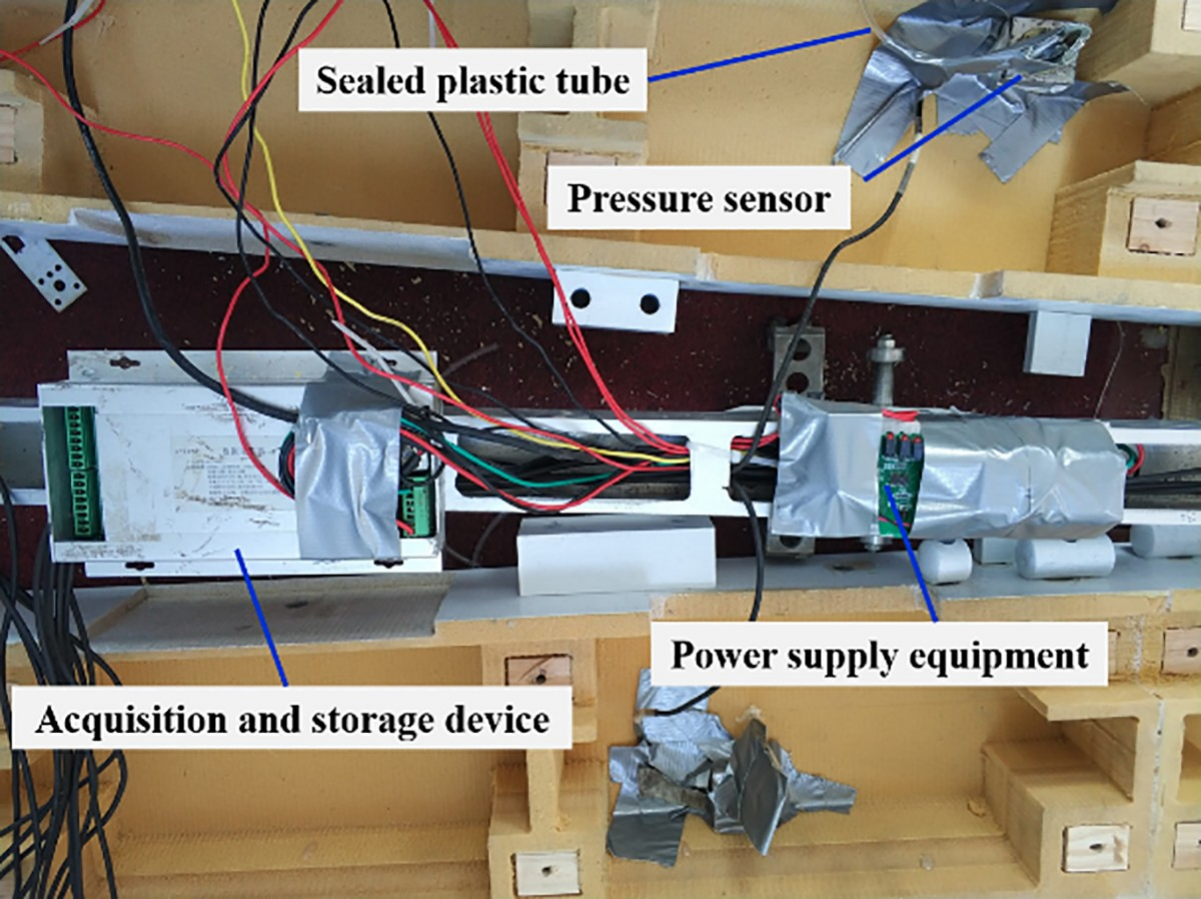
System for measuring pressure on the train model's surface.

Fig 5 shows the measurement system used in this study to measure the pressure on the surface of the model train, which consists of three main components: (1) pressure sensors, (2) power supply equipment, and a (3) data acquisition and storage device. The train was subjected to a large impact during acceleration and deceleration. Each component was taped to prevent loosening, so some components may not be clear in Fig 5, especially the power supply equipment.

Pressure measurement holes 1.5 mm in diameter were drilled perpendicular to the train model's surface. The pressure sensor was affixed to the model train’s inner surface. A hollow plastic tube with inner and outer diameters of 1.0 and 2.0 mm, respectively, was used to connect the interior pressure sensor and the surface pressure measurement hole. The plastic tube connecting the pressure sensor and measurement hole should be as short as possible. This is because, as tube length increases, response time increases, distorting the relationship between pressure and time. Also, a long tube can act as a filter, which distorts the pressure value.

The power supply equipment included a chargeable battery and a voltage regulator, which provided a stable voltage of 5 v to the sensors and the acquisition and storage device. The acquisition and storage device was installed in a metal box affixed to the steel beam. Any voids inside the metal box were filled with a foam material to prevent damage to the acquisition and storage device during the train model’s acceleration and deceleration.

The measurement method used to measure pressure changes on the tunnel model’s surface was similar to the measurement method used to measure pressure changes on the train model’s surface. Pressure measurement holes were drilled perpendicular to the tunnel model’s surface and sensors were fixed on its external surface. The hole was sealed with glue to keep the tunnel tight.

The train speed was measured with a photoelectric sensor at the tunnel entrance. As the train head and tail passed by the photoelectric sensor, it produced a voltage pulse; the train speed was acquired by dividing the length of the train model by the time interval between the two voltage pulses. In this study, the difference between measured speed and the target value was within ±2%, and we only selected replicates for which the error was within 1% for the next analysis. When the train entered the tunnel, the train head, main body, and tail generated pressures of different natures and amplitudes. To fully develop the aerodynamic pressure, the minimum tunnel length should not be shorter than 20 m in full scale test. [35]. Therefore, for the 1/10 scaled test, the tunnel length should be longer than 2m. The tunnel length was Ltu = 23.6 m in this study, which is longer than 2m.

Both the pressure at the train surface and the pressure at the tunnel wall were measured by differential pressure sensors in this study, with accuracy within ±5% [28]. We only selected the test with the error within 1% for the next analysis. The slipstream in the tunnel was measured with a five-hole probe. All data was retrieved by a computer for analysis. As the model was scaled down, the collected signal time base was reduced. For the 1/10-scale model in this study, the time bases of all of the pressure waves and slipstreams of the model train were 10 times faster than the time bases of the full-scale train. Since the pressure of the moving model test changed very quickly, the sensing measurement channel was required to have a high response speed. The data sampling frequency of the measurement system was 5 kHz. The data set was smoothed using a first low-pass Butterworth filter with a cut-off frequency of 0.005 s [31–32, 35].

## Discussion of results

### Experimental and numerical calculation results validation for slipstream

**Fig 6:**
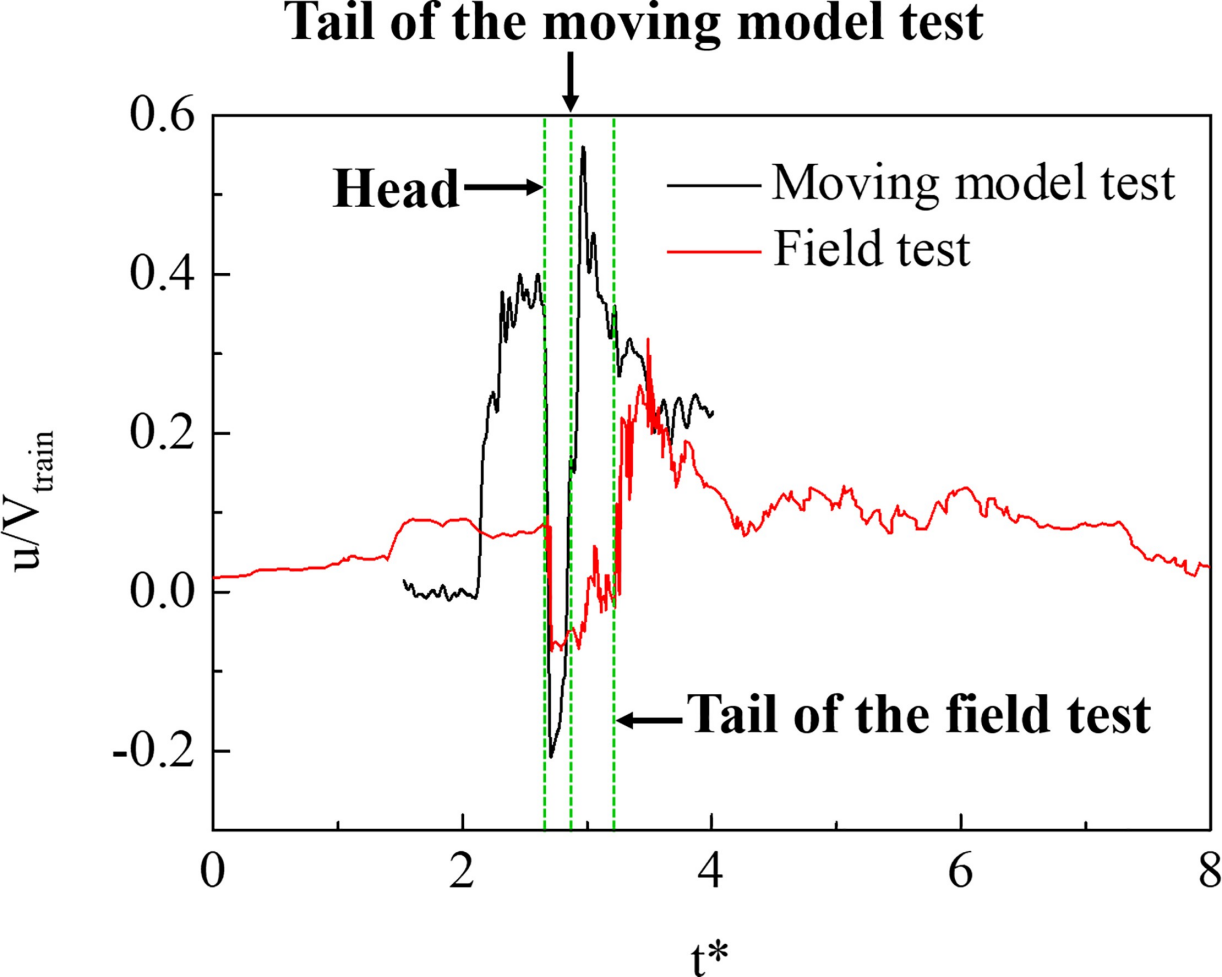
Comparison between the model test and the field test.

In order to verify the reliability of the moving model test, a field test on the aerodynamic effect in a tunnel was carried out. We now evaluate the results of the slipstream test between the model test and the field test, as shown in Fig 6. The law of variation of the slipstream was basically the same, but the amplitude of the two conditions was very different. First, the dynamic model test model consisted of a three-car multiple unit, and the trains in the field test consisted of an eight-car multiple unit. Therefore, when the train head passed the measurement point, the tail of the train in the field test, passed the measuring point later than in the dynamic model test. Second, the blockage ratio of the field test is smaller than that of the dynamic model test, which results in a smaller amplitude of the field test after a dimensionless for slipstream.

**Fig 7:**
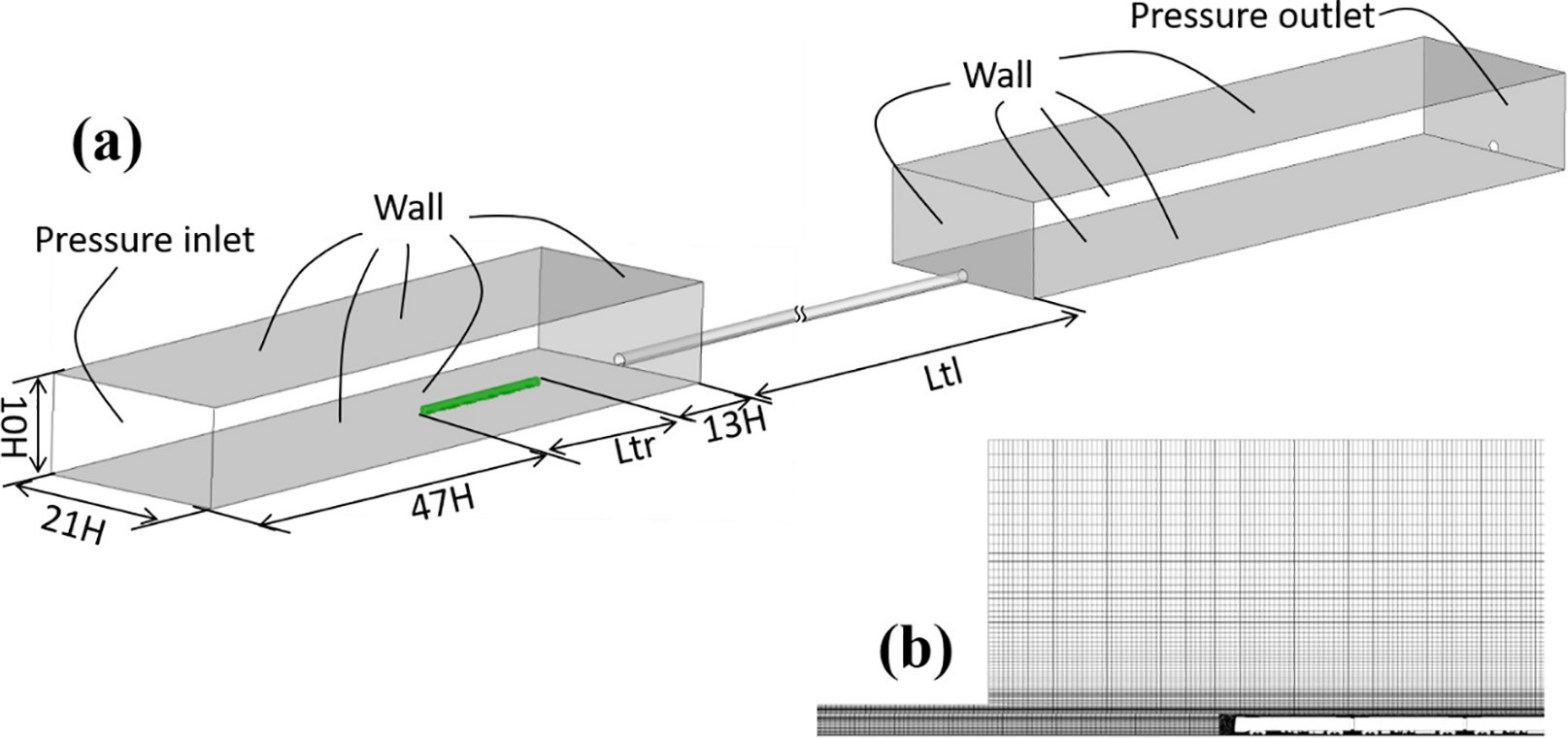
Computational domain and mesh.

**Fig 8:**
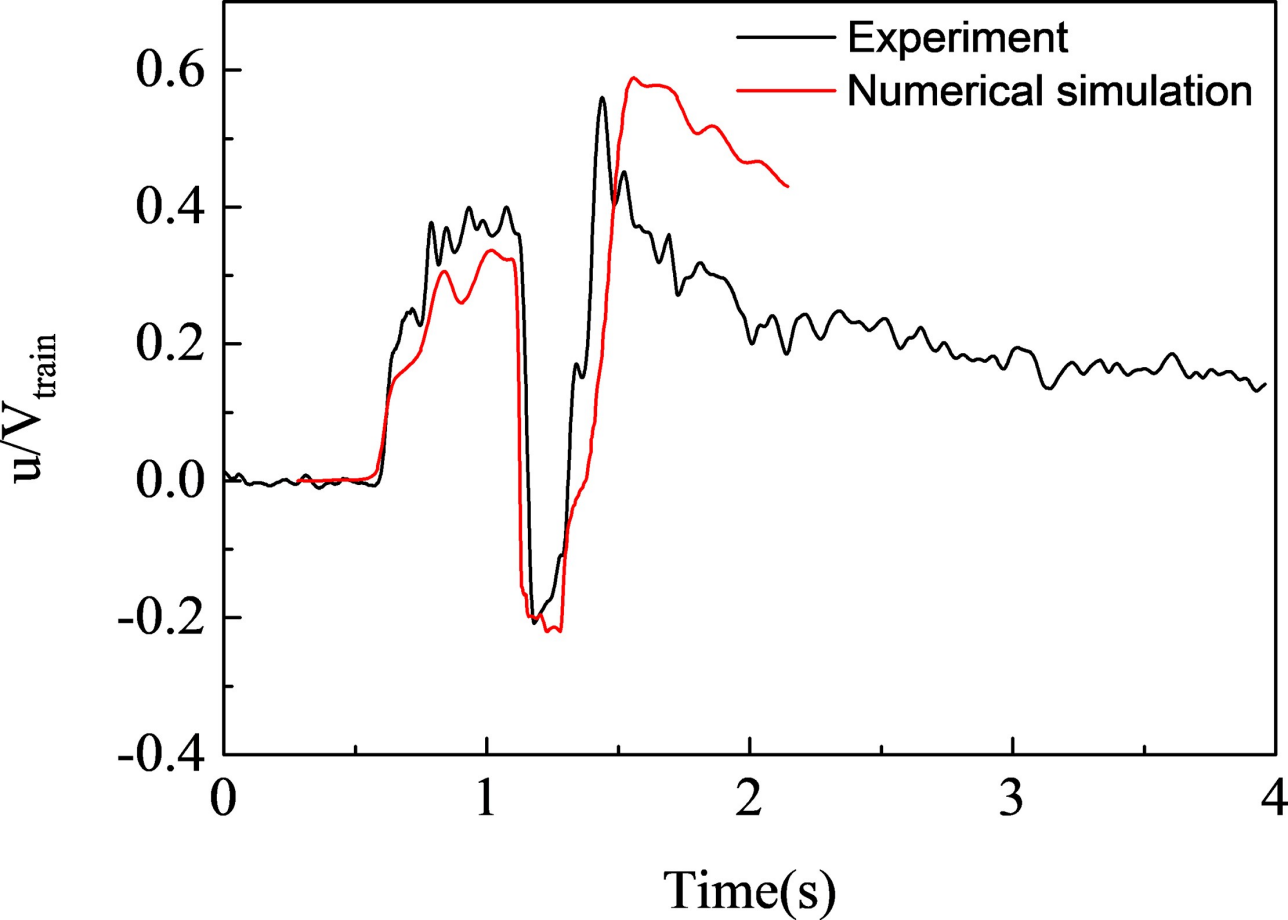
Slipstream along the x-direction.

To further analyze the slipstream amplitude, we built a 1/10 train-tunnel model to simulate a train passing through a tunnel at a speed of 120 km/h. The calculation domain, boundary conditions and mesh are shown in Fig 7, and the slipstream along the x-direction is shown in Fig 8. In general, the results of the dynamic model test are basically consistent with the numerical simulation results, and the slipstream amplitude error is about 5%. In addition, the trend of slipstream reduction after train passing is different, which may be caused by actual environmental factors.

### Repeatability tests

**Fig 9:**
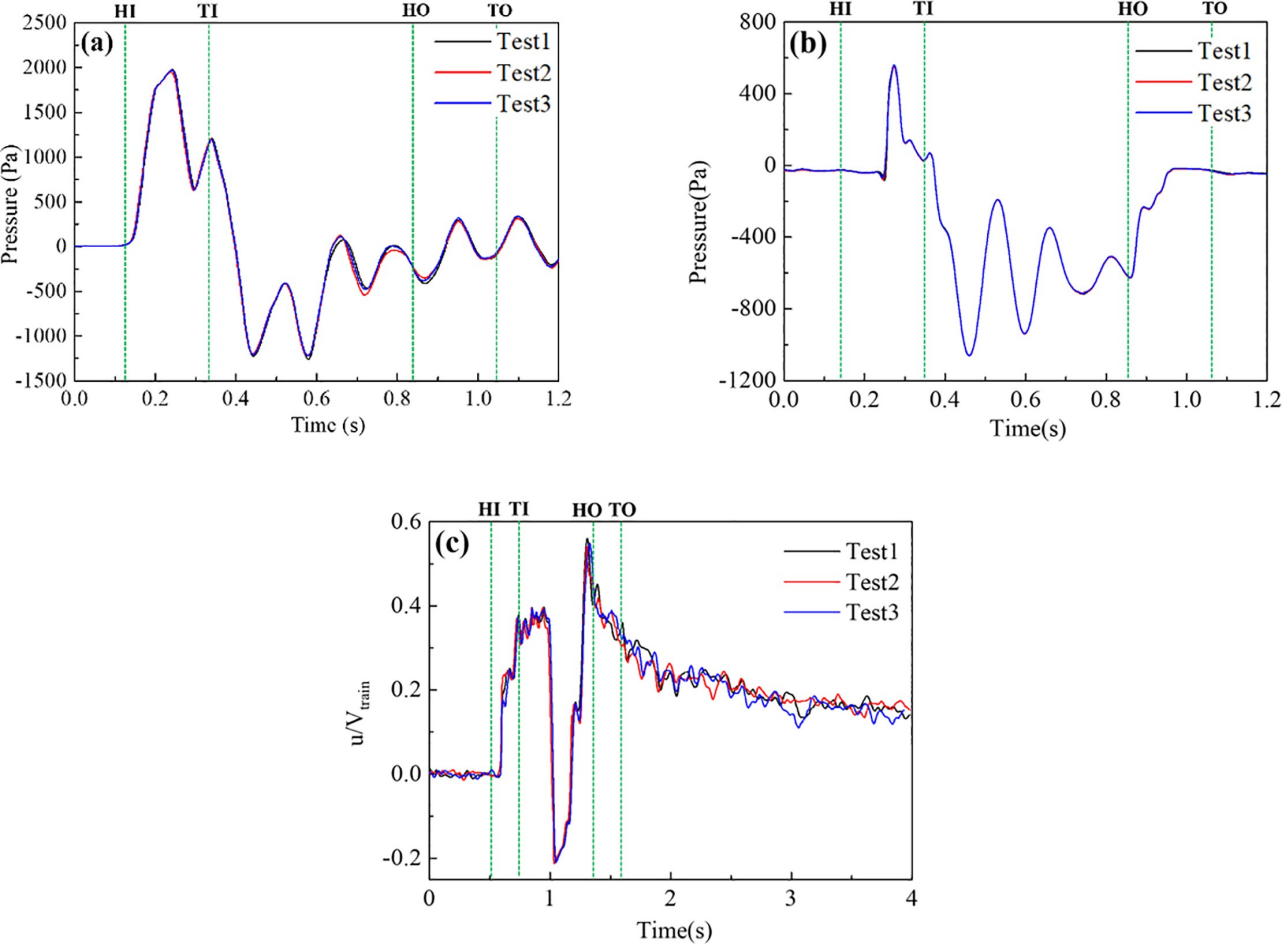
Repeatability of the test results. (a) Pressure at T5 on the tunnel wall, (b) Pressure at H5 on the train surface, (c) Slipstreams at T5.

Repeatability tests were conducted 15–20 times for each test condition. Fig 9 shows the comparison between the most accurate results of the three repeatability tests selected when the train was traveling through the tunnel at 120 km/h. The actual speed of the train entering into tunnel was 119.3 km/h、120.2km/h、119.6km/h, respectively. Fig 9(A), 9(B) and 9(C) shows the time-history curves for the pressure at the tunnel wall, the pressure at the train surface, and the slipstream in the tunnel. It can be concluded from the figures that the results of the three tests for the pressure at the tunnel wall and the pressure at the train surface coincided well. This was because the pressure was primarily caused by compression and expansion waves influenced by the train speed. As shown in Table 1, the largest difference between the pressure amplitudes at the tunnel wall was 1.1% and the largest difference between the pressure amplitudes at the train surface was 1.2%.

**Table 1:** Pressure (T5) and Slipstream (T5) value of the repeatability test.

Related variables	Pressure of the tunnel wall			Pressure of the train surface			Slipstream velocities		
	Test1	Test2	Test3	Test1	Test2	Test3	Test1	Test2	Test3
P_max_	1750	1733	1769	554	551	569	0.552	0.549	0.574
P_min_	-1030	-1078	-1036	-1059	-1062	-1073	-0.209	-0.207	-0.208
ΔP	2780	2811	2805	1623	1613	1642	0.761	0.756	0.782
Diff./Ref.(%)	/	-1.1	-0.9	/	-0.6	-1.2	/	-0.4	2.7

Compared with the pressures, the time-history curves for the slipstream in the tunnel, as shown in Fig 9(C), did not coincide as well. Table 1 shows that the biggest difference between the amplitudes of the slipstream was 2.7%. This was because the turbulence caused by the moving train was complex, especially in the flows caused by the train wake. In the actual moving model test, it was impossible to completely remodel the airflow in tunnel. For the test, u denotes the longitudinal component of the slipstream velocity, V_train_ denotes the velocity of the train, P_max_ denotes the maximum pressure or the maximum slipstream velocity, P_min_ denotes the minimum pressure or the minimum slipstream velocity, and ΔP denotes the pressure variation amplitude or the slipstream velocity amplitude. The time when the train first enters the tunnel is marked as HI. TI, HO, and TO represent the time when the end of the train enters the tunnel, the time when the train engine leaves the tunnel, and the moment when the end of the train leaves the tunnel.

### Impacts of the airshaft on the transient pressure and the slipstream

**Fig 10:**
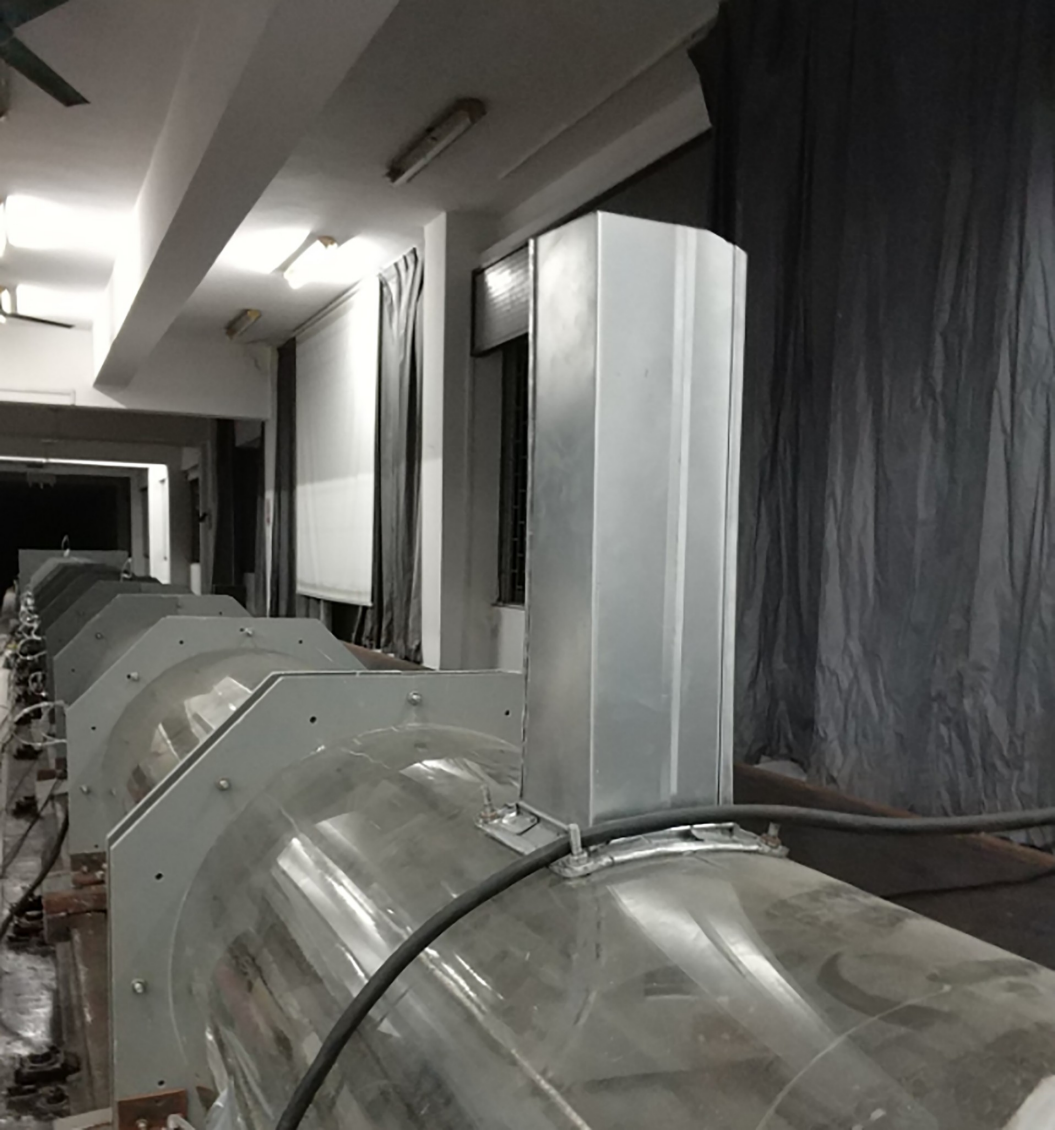
Tunnel-airshaft schematic diagram.

This section discusses the airshaft added in the middle of tunnel to study the impacts of airshafts on the transient pressure and slipstream. The length and width of the airshaft cross section were both 0.2 m; the ratio of the airshaft area to the tunnel area was 0.18. The tunnel-airshaft structure is shown in Fig 10.

**Fig 11:**
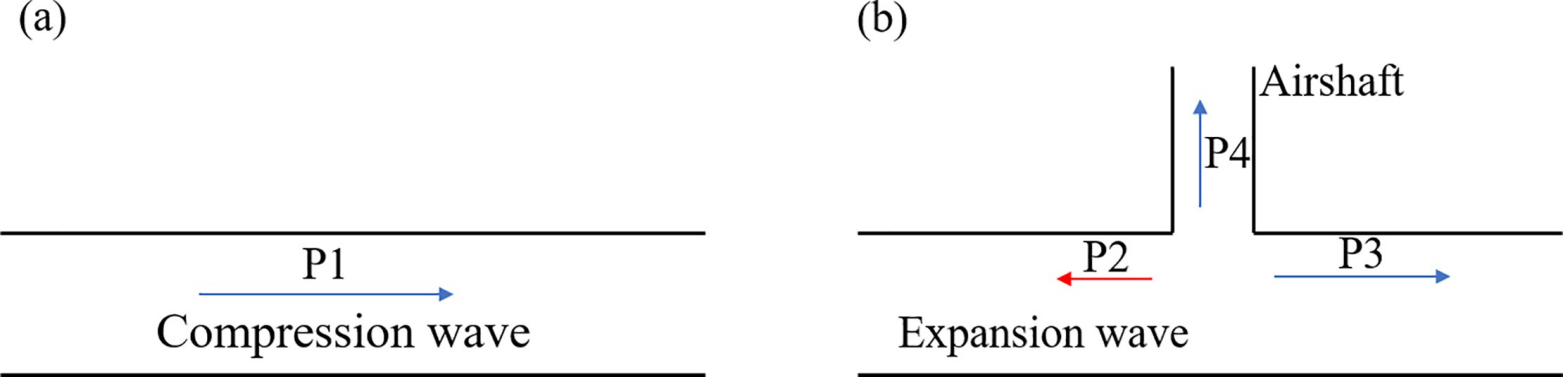
Wave propagation and reflection with and without an airshaft. (a) without an airshaft, (b) with an airshaft.

Having a central airshaft in the tunnel made the pressure wave propagation more complex. As shown in Fig 11(A) and 11(B), P1, P2, P3 and P4 denote incoming compression wave from the main tunnel, reflected and transmitted into the main tunnel and transmitted into the airshaft, respectively [2].The pressure wave was reflected not only at both ends of tunnel, but also at the airshaft, where a new pressure wave was generated. The pressure wave reflected at the airshaft was opposite the original pressure wave (the original pressure wave was a compression wave and the reflected wave was an expansion wave). When the pressure wave P1 reached the airshaft, it could be divided into three parts. One part became the reflected wave P2 at the airshaft and was transmitted in the opposite direction; a second part P4 was transmitted outwards along the airshaft; and the last part P3 was transmitted in the original direction. The intensity of P3 was smaller than that of P1 [2].

#### Impact on the transient pressure

Table 2 provides the values of ΔP at point T5 on the tunnel wall with and without an airshaft for all measurements, as well as the calculated means, the standard deviations, and the permissible characteristic ΔP for the two cases (ΔP_95%_). σ with airshaft is bigger than without airshaft, which may be due to more complicated pressure wave propagation. Note that only the value of 2716 for measurement 5 without airshaft is not within the range of the permissible characteristic (ΔP_95%_). In this way, the test results with and without an airshaft are reliable.

**Table 2:** ΔP at point T5 on the tunnel wall with and without an airshaft.

Measurement	With airshaft (Pa)	Without airshaft (Pa)
1	1783	2780
2	1746	2801
3	1801	2805
4	1789	2811
5	1779	2716
6	1792	2816
7	1820	2846
8	1809	2803
9	1801	2764
10	1778	2832
11	1763	2763
12	1795	2735
13	1749	2750
14	1753	2799
15	1771	2786
16	1786	2779
17	1806	2793
18	1771	2783
19	1796	2769
20	1779	2794
Mean ($\Delta \bar{P}$)	1783	2786
St. Dev. (*σ*)	33	28
$\Delta P_{95\%}(\Delta \bar{P}\pm 2\sigma )$	(1717, 1849)	(2730, 2842)

**Fig 12:**
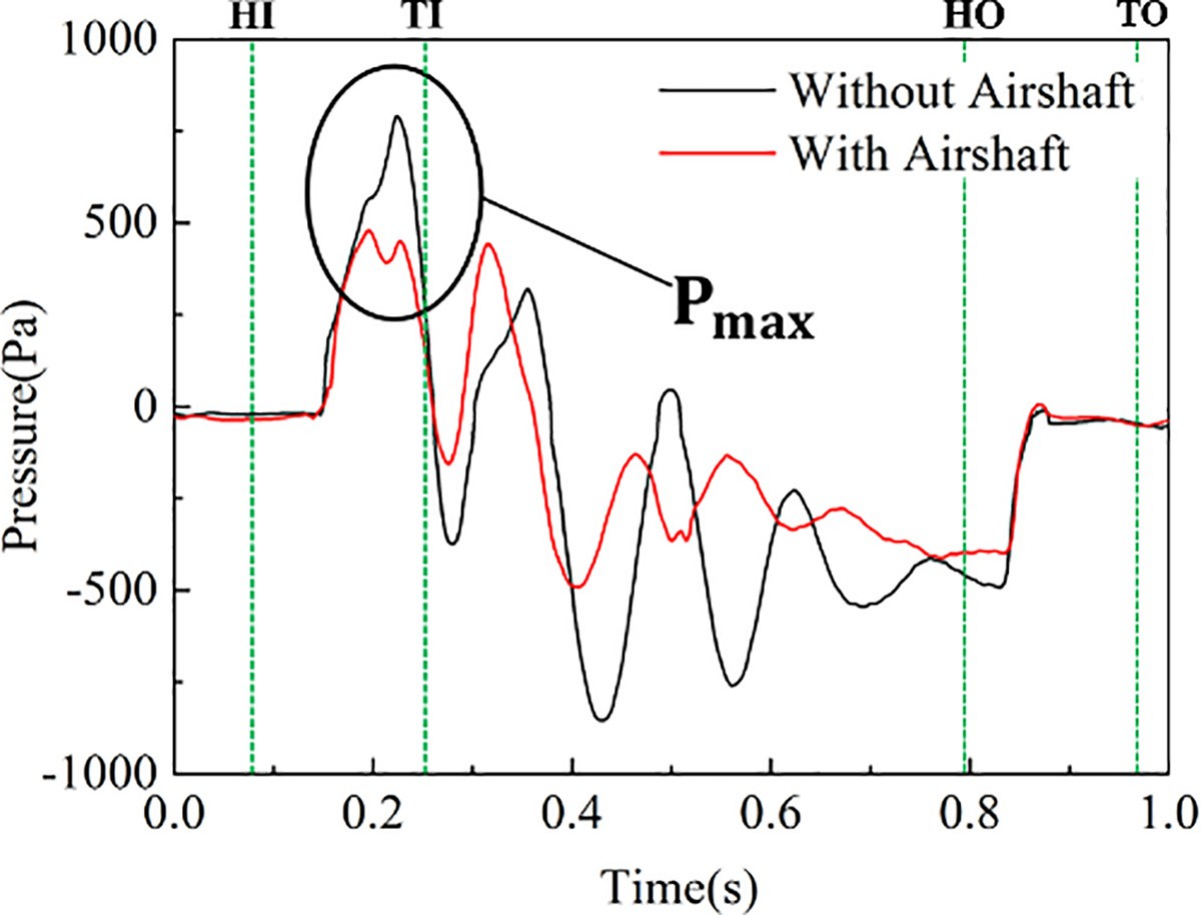
Pressures at H4 on train surface.

The pressure on the train surface with and without an airshaft is shown in Fig 12. It can be concluded from the figure that the presence of an airshaft weakens the positive and negative pressure peaks on the train surface. The difference in P_max_ comes from the reflection of initial compression wave at the airshaft. The reflection is an expansion wave transmitted opposite train motion; it dropped the pressure along the train surface early. However, because the initial compression wave generated by the train entering the tunnel is reflected at the other end of the tunnel when there is now airshaft, the P_max_ for pressure without an airshaft dropped later.

**Fig 13:**
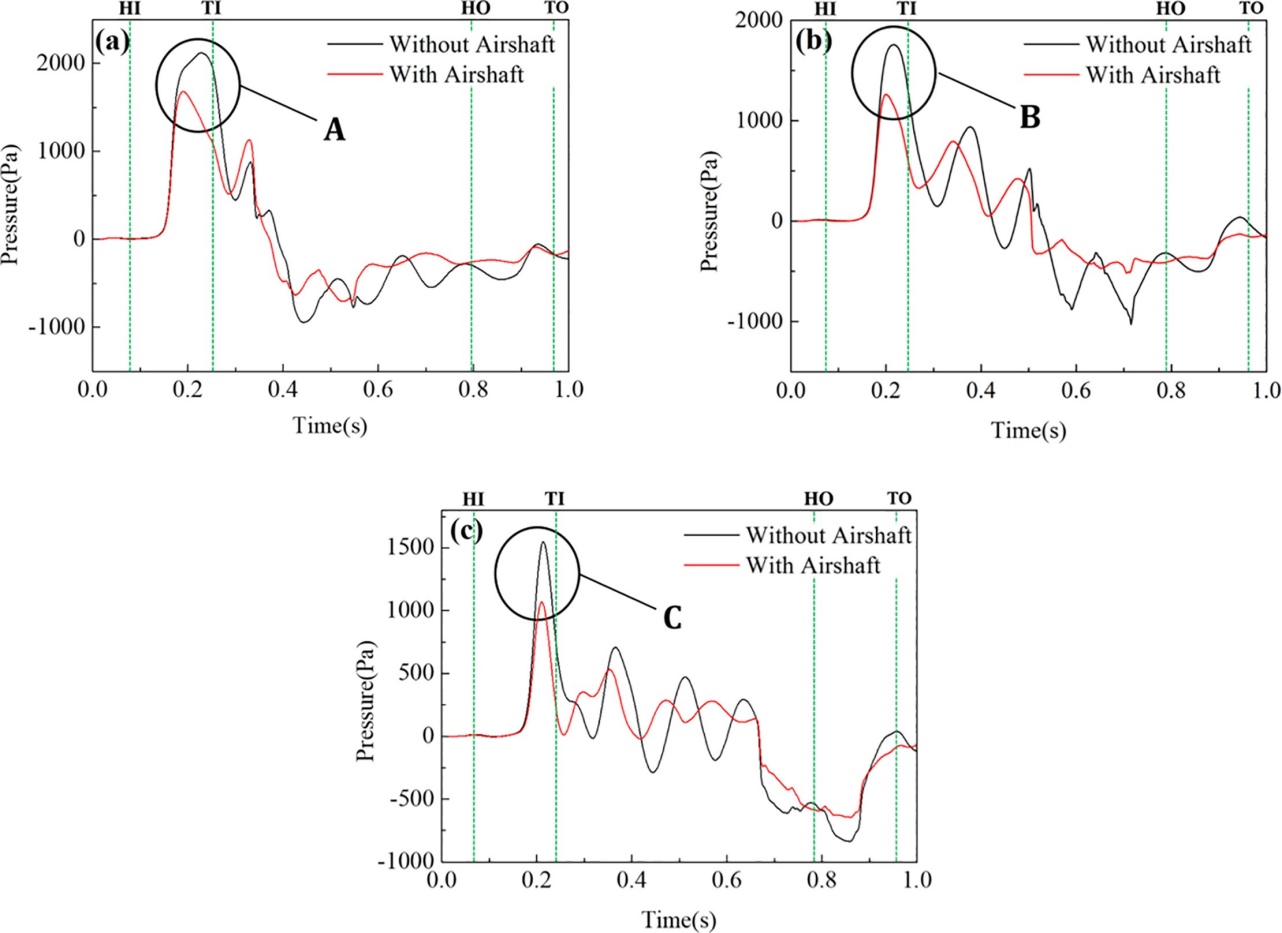
Variation of the pressure at different measuring points on the tunnel wall. (a), (b) and (c) show the measuring points located before the airshaft (T3), at the airshaft (T5) and after the airshaft (T7), respectively.

Fig 13(A)–13(C) shows the pressure with and without an airshaft ahead of the airshaft location, at the airshaft, and after the airshaft. The pressure peaks before the airshaft, at the airshaft, and after the airshaft are labeled A, B, and C, respectively. All values dropped after the installation of the airshaft, but for different reasons. At A, the interference of the expansion wave reflected at the airshaft and the compression wave generated by the train entering the tunnel led to the pressure drop. At point B, the complex interactions between P2, P3 and P4 combined to reduce the pressure. At C, the compression wave (P3 in Fig 11) was weakened after passing by the airshaft.

**Fig 14:**
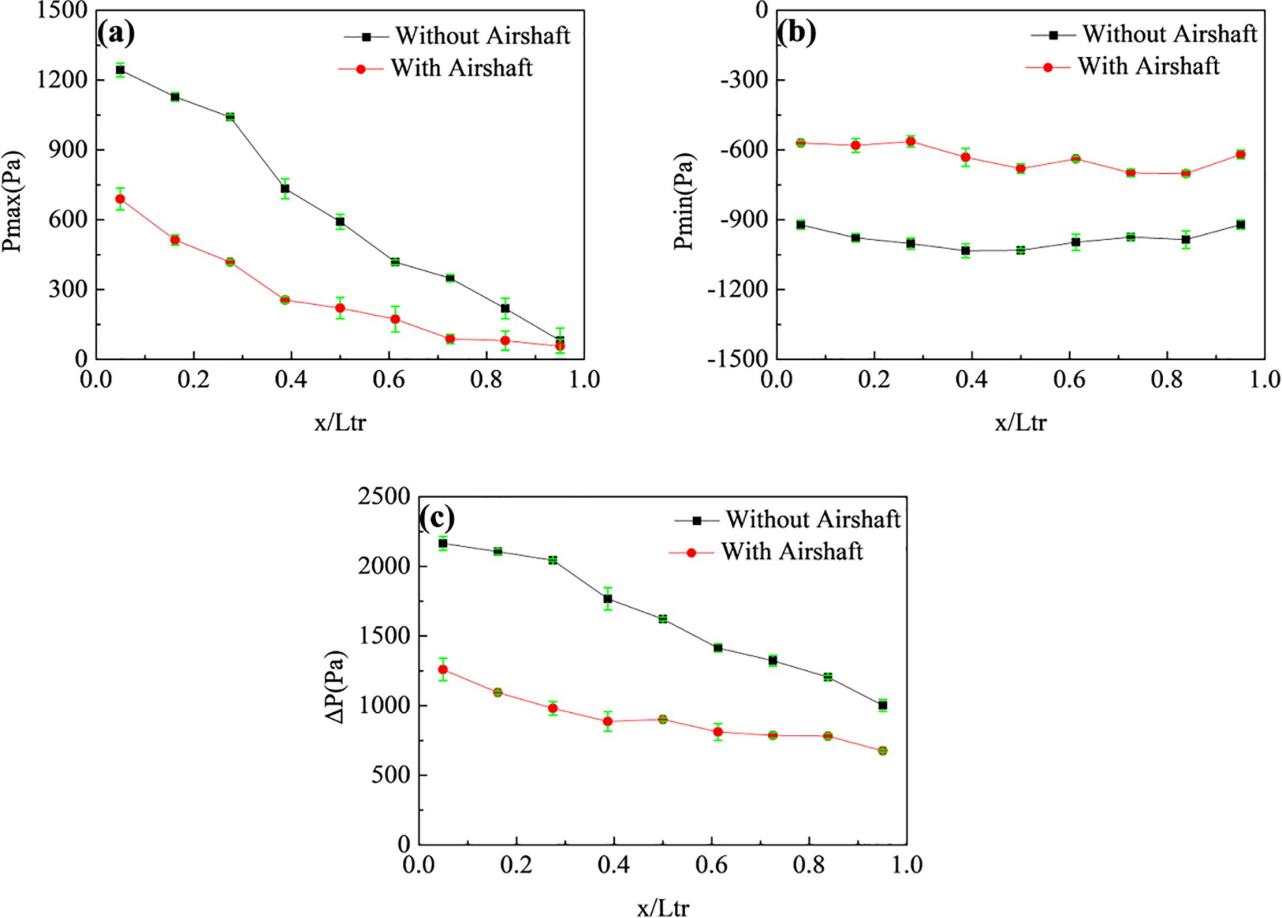
Pressures at different points on the train surface with and without an airshaft. (a), (b), and (c) show P_max_, P_min_, and ΔP, respectively.

Fig 14A–14C shows the P_max_, P_min_, and ΔP at different points on the train surface with and without the airshaft. Without going into details, the mean is a way of summarizing data and offering a best guess at what the true value of the dependent variable value is for that independent variable level. The representative value of Pressure $\bar{P}$ is obtained as the mean value of multiple running, i.e. $\bar{P}=\sum_{1}^{n}P_{i}/n$. According to the mean value, we calculate the standard error after getting a standard deviation.

**Fig 15:**
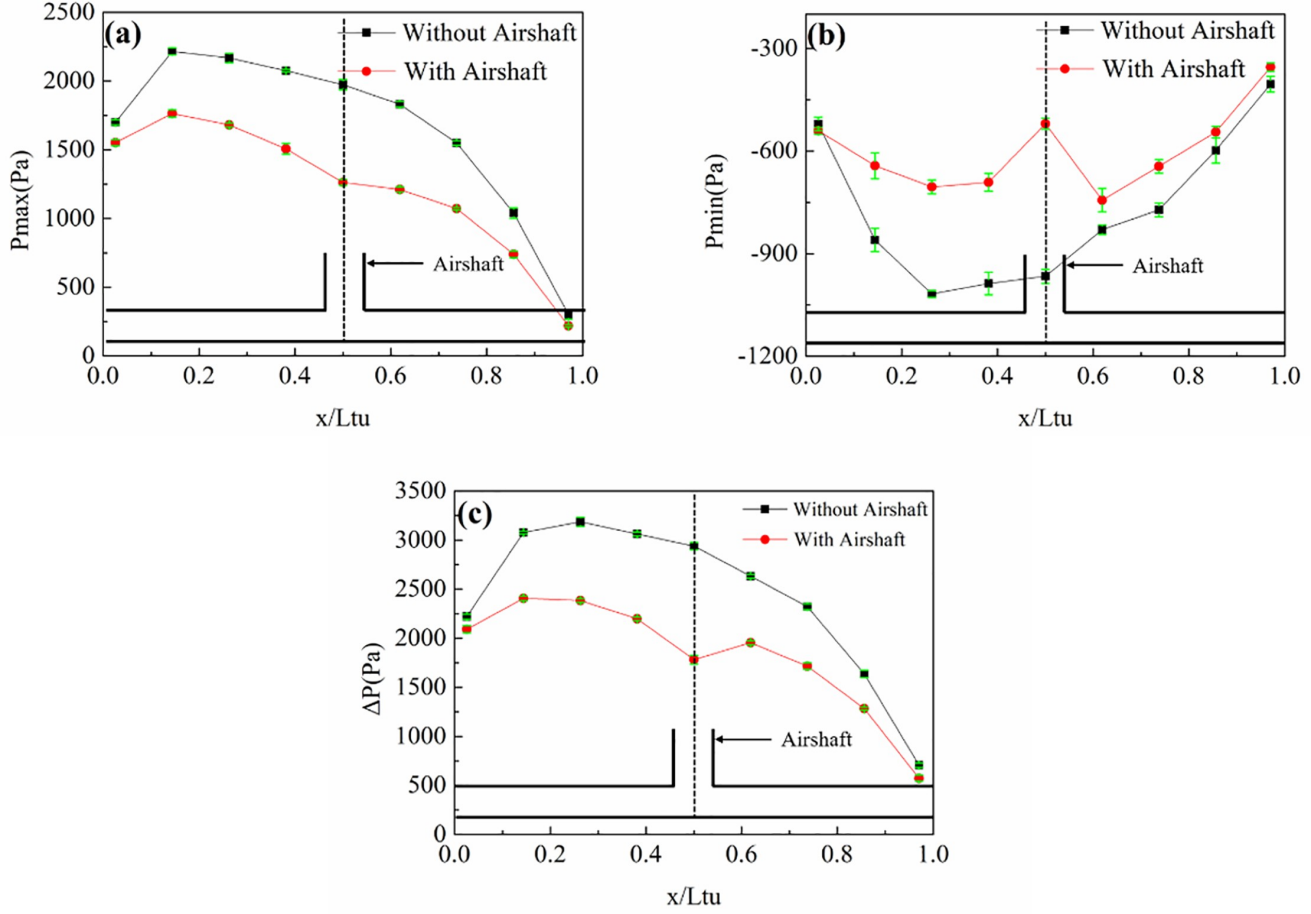
Pressures at different points on the tunnel wall with and without an airshaft. (a), (b), and (c) show P_max_, P_min_, and ΔP respectively.

According to Fig 14, the airshaft does not change the pressure trends along the train length, but it can relieve the pressure on the train surface. The relief effect at the head car was especially significant, and the pressure maximum and amplitude decreased by 44.5% and 41.8%, respectively. Fig 15A–15C shows P_max_, P_min_, and ΔP at different points on the tunnel wall with and without an airshaft. In contrast to the pressure on the train surface, the airshaft significantly changes the pressure trend along the tunnel length. P_max_, P_min_, and ΔP at the point in the middle of tunnel decreased with an airshaft because the compression and expansion waves spread outward from the tunnel at the airshaft, decreasing their energy. Simultaneously, the pressure wave generated a reflected wave that interfered with the other pressure waves, to achieve a good pressure relief effect. P_max_, P_min_, and ΔP in the middle of the tunnel decreased by 36.0%, 39.4%, and 46.2%, respectively. Additionally, the pressure reduction from the middle to both ends of the tunnel decreased, so that the impact of the airshaft on the tunnel entrance and exit pressures could be essentially ignored. This was because the energy of the reflected wave was small and weakened as it traveled through the tunnel.

Table 3 shows the comparison of the pressures at different point(before airshaft: x/L_tu_ = 0.26, at airshaft: x/L_tu_ = 0.5, after airshaft: x/L_tu_ = 0.72)with and without an airshaft. P_max_, P_min_, and ΔP for the points at the airshaft dropped by 28.1%, 49.5%, and 36.0%, respectively. These were much greater than the drops for the other positions, indicating that there was a pronounced pressure relief effect around the airshaft.

**Table 3:** Pressure values at different points of the tunnel before and after including an airshaft.

Measuring points	Without airshaft			With airshaft			Reduction		
	P_max_	P_min_	ΔP	P_max_	P_min_	ΔP	P_max_	P_min_	ΔP
Before airshaft	2180	-1057	3237	1680	-705	2385	22.9%	33.3%	26.3%
At airshaft	1756	-1030	2786	1263	-520	1783	28.1%	49.5%	36.0%
After airshaft	1407	-760	2166	1072	-645	1716	23.8%	15.1%	20.8%

#### Impacts on the slipstream

**Fig 16:**
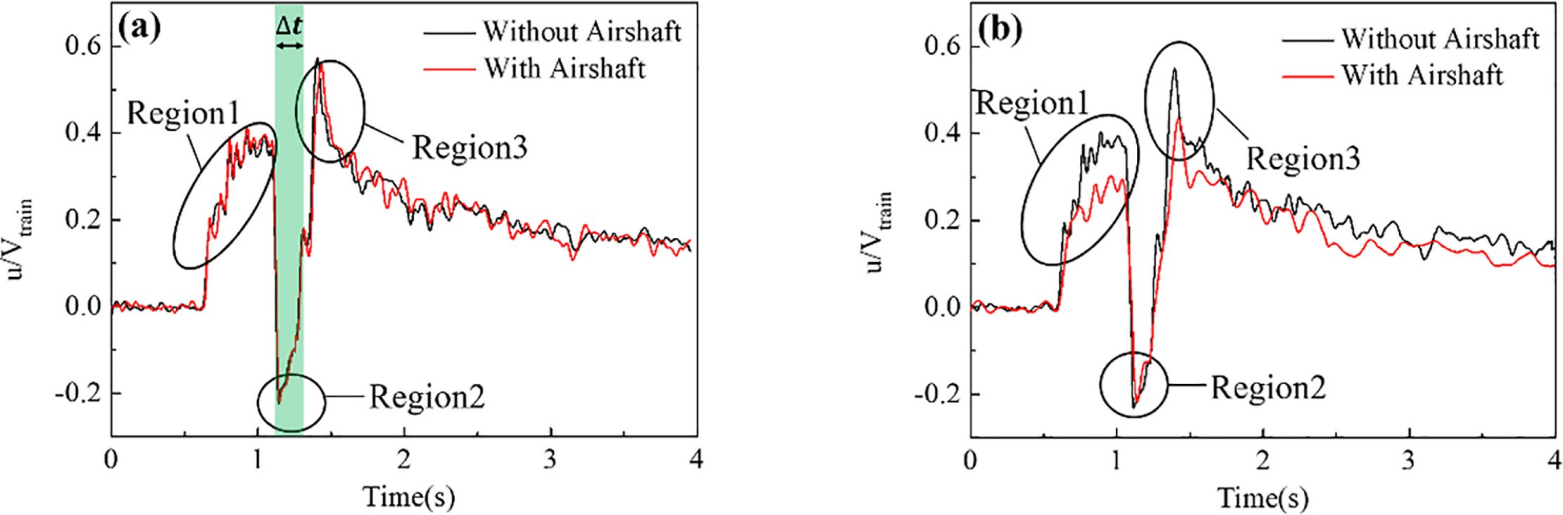
The slipstreams at different points with and without an airshaft. (a) and (b) show the measuring points before (T3) and after (T7) airshaft, respectively.

Fig 16 shows the slipstream at different points with and without an airshaft. The impact of the airshaft on the slipstream at different points was quite different. At first, Fig 16(A) is used as an example to describe the process of slipstream generation. The green region denotes the time period when the train was passing by, Δt = 0.21s. When the train entered the tunnel, the compressed air at the nose generated a piston effect and increased the slipstream speed in Region 1. After the train had fully entered the tunnel, the slipstream speed almost fluctuated around 0.38, as shown in the second half of Region 1. As the train head passed by the measuring points, both the slipstream direction and the value changed suddenly, as shown in Region 2. As the train tail passed by the measuring point, the slipstream velocity and the direction changed again, decreasing rapidly to 0 and then increasing in the positive direction. This happened because the space between the train surface and the tunnel wall decreased, cause the air to flow relatively slowly opposite the direction of train travel. When the train passed the measuring points, the train wake increased the slipstream speed to its maximum in the same direction as train travel, as shown in Region 3. With the train driving away, the wake effect weakened gradually and the slipstream speed decreased gradually.

It can be concluded from Fig 16(A) that the trends for the slipstream before and after the airshaft were essentially the same, with the airshaft having little impact at all on the slipstream before the airshaft. However, the slipstream at the points after the airshaft was quite different from that without an airshaft, as shown in Fig 16(B). Region 1 refers to region in which the slipstream decreased from the original 0.38 to about 0.26 as the train passed the measuring point. This decrease in intensity occurred because the slipstream generated by the train entering the tunnel lost part of its energy as it passed the airshaft. The slipstream speed in Region 2 was essentially not impacted by the airshaft. The slipstream speed in Region 3 decreased from 0.55 to 0.43, because the train wake was impacted by the airshaft, decreasing the slipstream intensity.

### Impacts of the speed on the transient pressure and the slipstream

#### Impacts on the transient pressure

**Fig 17:**
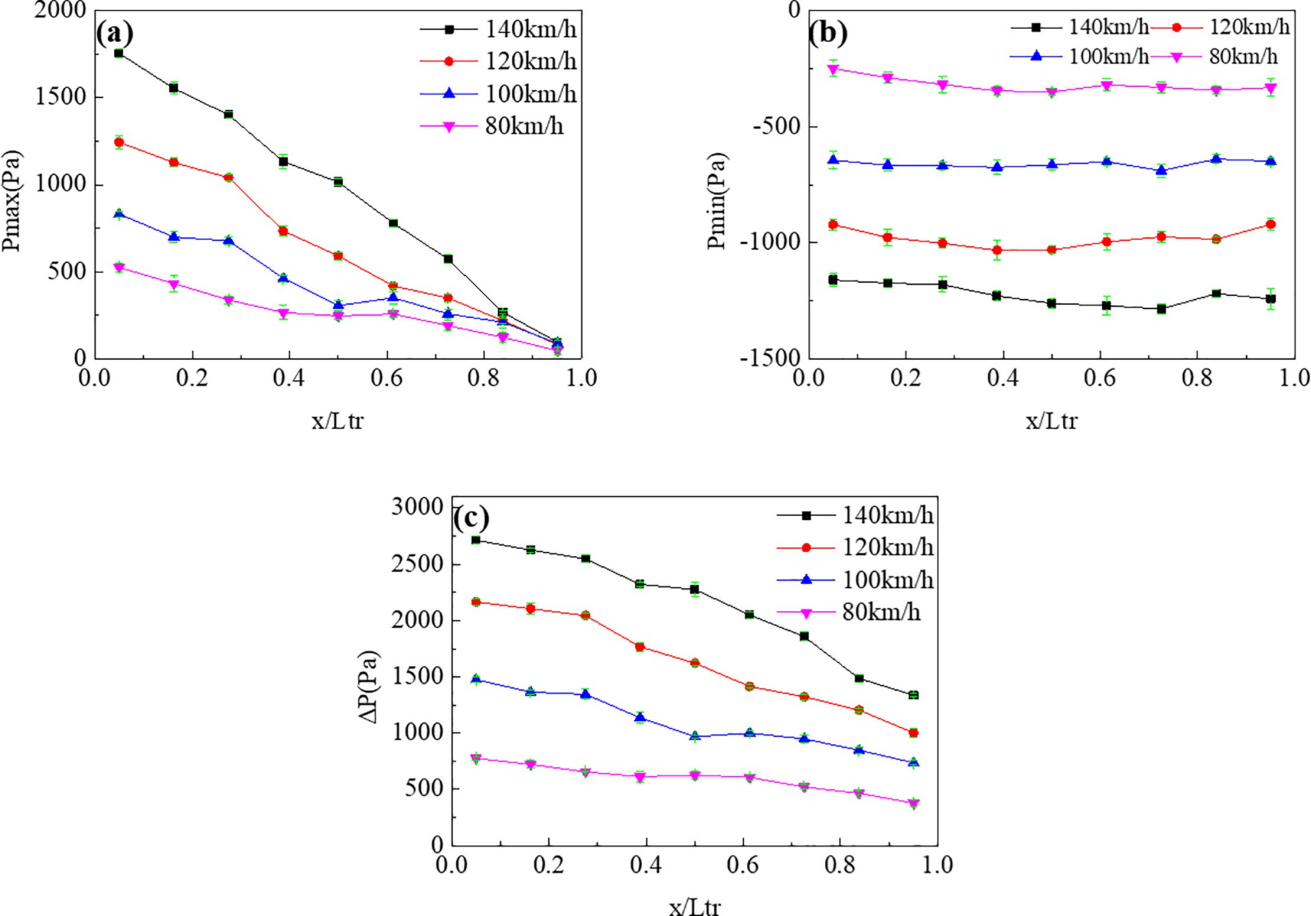
Pressure values at different points (z/H = 0.49) on the train surface under different speeds. (a), (b), and (c) show P_max_, P_min_, and ΔP, respectively.

Fig 17A–17C shows P_max_, P_min_, and ΔP at different speeds grades. The pressure variation trends on the train surface did not change. P_max_ and ΔP on the train surface decreased from the head to the tail along the train length, but P_min_ on the train surface remained essentially the same. However, the pressure values noticeably increased with increasing speed. It is worth noting that the growth rates also increased with increasing speed. For instance, the P_max_ of point at x/L_tr_ = 0.05 was 524Pa、830Pa、1242Pa、1754Pa with the speed increasing from 80km/h to 120km/h. Pressure increased by 306Pa, 412Pa, 512Pa respectively.

**Fig 18:**
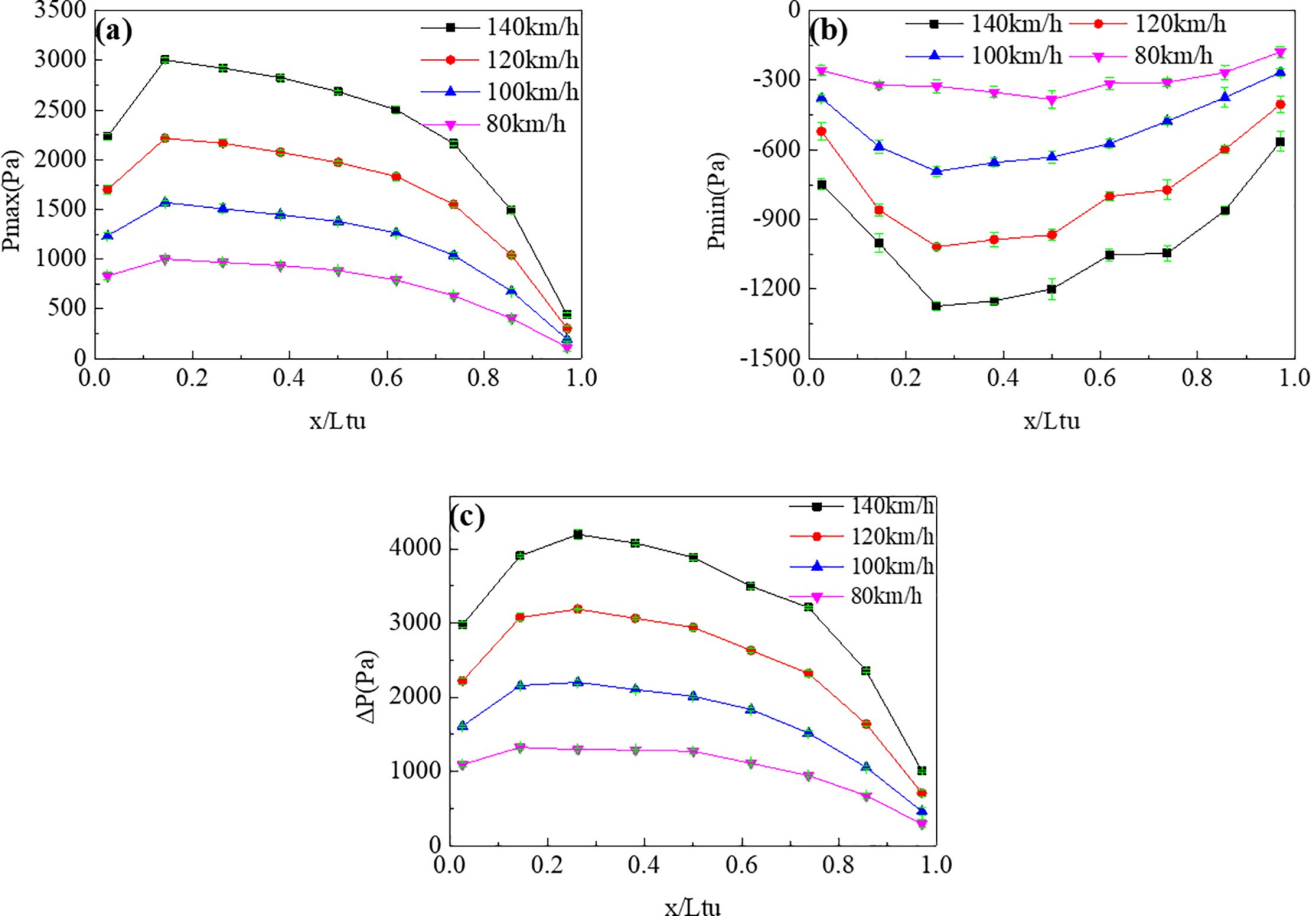
Pressure values at different points (z/H = 0.90) on the tunnel wall with different train speeds. (a), (b), and (c) show P_max_, P_min_, and ΔP, respectively.

Fig 18A–18C shows P_max_, P_min_, and ΔP at different points on the tunnel wall at different speeds. The change in speed did not change the variation trends of the pressures on the tunnel wall. For instance, P_max_, P_min_, and ΔP all decreased from the front to the middle part of the tunnel for both sides. With increasing train speed, the energy of the compression waves and expansion waves also increased, leading to both increasing pressures value and growth rates.

In Fig 17A and Fig 18A, through the comparison of the pressure value, it is found that the maximum P_max_ value at the tunnel wall is significantly larger than that of the train surface because of the piston effect. The P_min_ value at the tunnel wall was almost the same as that on the train surface, indicating that the minimum pressure in the tunnel was caused by the train wake.

**Fig 19:**
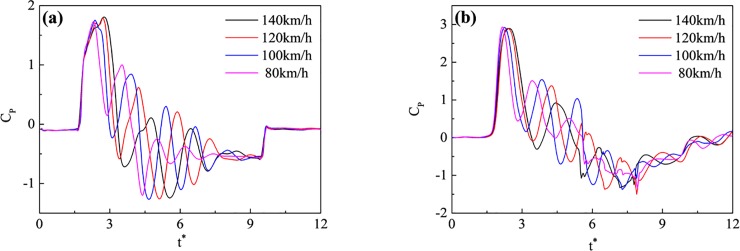
Curves for the pressures at points on the train surface and the tunnel wall with time. (a) Points at H4 on the train surface; (b) Points at T4 on the tunnel wall.

To further compare and analyze the impact of speed on the pressure wave, a dimensionless method was applied to normalize time t and pressure p. The dimensionless time is shown as the X-axis in Fig 19, and the dimensionless formulas are shown below:

$$t^{*}=tV_{train}/(10H),$$ (1)

$$C_{p}=P/(0.5\rho V_{train}^{2}),$$ (2)

Where t* denotes the dimensionless time, t denotes the actual time, C_P_ denotes the pressure coefficient, P denotes the pressure, and ρ denotes the air density, (1.225 kg/m^3^).

The dimensionless pressure curves are shown in Fig 19. With increasing speed from 80 km/h to 140 km/h, the largest differences between the maxima, minima, and amplitudes of the pressure coefficients on the train surface were 3.3%, 3.8%, and 3.6%, respectively. The largest differences between the maxima, minima, and amplitudes of the pressure coefficients on the tunnel wall were 1.3%, 4.3%, and 2.2%, respectively. This indicated that pressure coefficient on the train surface and tunnel wall did not change with the speed. However, the pressure curves were quite different. This was because the time and energy were generated by compression and expansion waves induced at different speeds, and the locations and times of constructive interference were different.

#### Impacts on the slipstream

**Fig 20:**
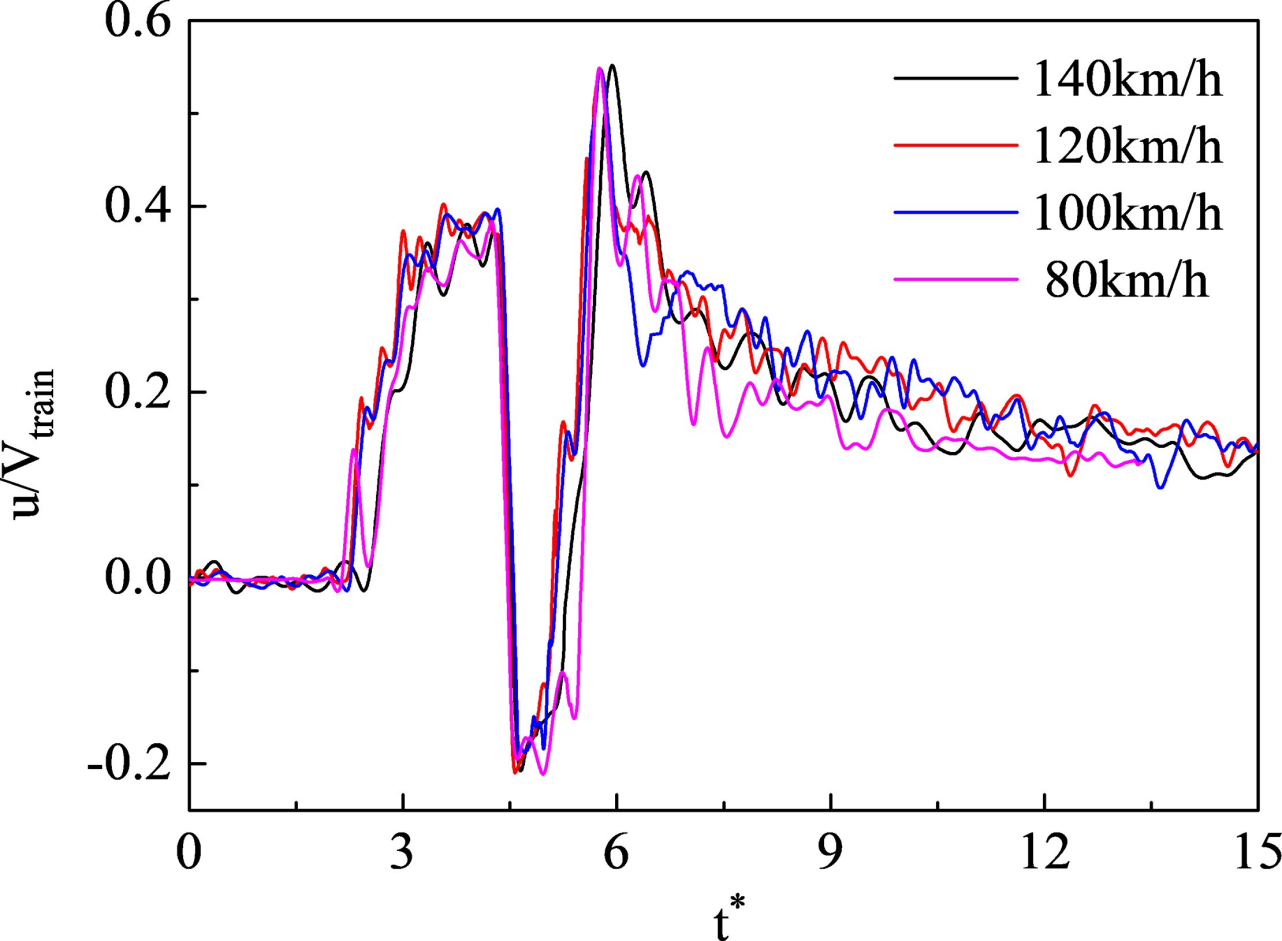
Curves for the slipstreams that changed with time(T5).

Fig 20 shows the slipstream at different speeds with a dimensionless x-axis as in Fig 19. It is evident that the waveform of the slipstream fluctuated with increasing train speed, due to uncertain factors such as complex turbulence induced by the train. However, even at different speeds, the trends describing the slipstream were essentially the same, especially at maximum u/V_train_.

## Conclusions

Using the moving model test platform and a 1/10 scaled train model, the effects of an airshaft and train speed on a metro train and tunnel aerodynamics were investigated. The train model traveled through a 23.6 m long tunnel model. Data obtained from multiple measurement points were analyzed, and the spatial distribution of transient pressure and slipstream were determined. The mechanism underlying the physical effect of the airshaft on the pressure is here explained. Based on the results presented herein, general conclusions were deduced as follows:

(1) A central airshaft has different pressure relief effects at different locations in a tunnel. These effects were most pronounced in the middle of the tunnel, where the pressure amplitude decreased by 36.0%. The impact of the airshaft on the tunnel entrance and exit pressure could be essentially disregarded. The slipstream was somewhat alleviated after an airshaft. The slipstream speed in Region 3 decreased from 0.45 to 0.36, but the slipstream speed in Region 2 was essentially not impacted by the airshaft.

(2) Increase of the train speed led to increases in the pressure on the train surface and the tunnel wall and an increase in the pressure growth rate. The dimensionless pressure curves were quite different. However, increases in speed were not found to change the pressure coefficients. The waveform of the slipstream fluctuated with increasing train speed, due to uncertain factors such as complex turbulence induced by the train. However, the trends describing the slipstream were essentially the same.

(3) When the train traveled through a tunnel, P_max_ and ΔP on the surface of the train decreased from the head to the tail along the train, but P_min_ on the surface of the train remained essentially the same. The pressure on the tunnel wall peaked as the train passed through the first half of the tunnel and decreased along the tunnel toward both ends. The slipstreams in Region 1, Region 2, and Region 3 had different causes, and the slipstream values were different. When the train passed the measuring points, the train wake increased the slipstream speed to its max (u/V_train_ = 0.55) in Region 3.

## Supporting information

S1 Appendix (DOCX)

S1 Data (XLSX)

## References

[1] Ogawa T., & Fujii K., 1997 Numerical investigation of three-dimensional compressible flows induced by a train moving into a tunnel. Comput. Fluids. 26(6), 565–585.

[2] Baron A., Mossi M., & Sibilla S., 2001 The alleviation of the aerodynamic drag and wave effects of high-speed trains in very long tunnels. J. Wind Eng. Ind. Aerod. 89(5), 365–401.

[3] Ricco P., Baron A., & Molteni P., 2007 Nature of pressure waves induced by a high-speed train travelling through a tunnel. J. Wind Eng. Ind. Aerod. 95(8), 781–808.

[4] Khayrullina A., Blocken B., Janssen W., & Straathof J., 2015 CFD simulation of train aerodynamics: slipstream conditions at an underground railroad passenger platform. J. Wind Eng. Ind. Aerod. 139, 100–110.

[5] Chu C. R., Chien S. Y., Wang C. Y., & Wu T. R., 2014 Numerical simulation of two trains intersecting in a tunnel. Tunn. Undergr. Space Technol. 42(5), 161–174.

[6] Cross D., Hughes B., Ingham D., & Lin M., 2015 A validated numerical investigation of the effects of high blockage ratio and train and tunnel length upon underground railway aerodynamics. J. Wind Eng. Ind. Aerod. 146(17), 195–206.

[7] Fu M., Li P., & Liang X. (2017). Numerical analysis of the wake development around a high-speed train in a double-track tunnel. Plos One. 12(3), e0175044 10.1371/journal.pone.0175044 28362835 PMC5376298

[8] Wang T., Wu F., Yang M., Ji P., & Qian B. (2018). Reduction of pressure transients of high-speed train passing through a tunnel by cross-section increase. J. Wind Eng. Ind. Aerod. 183, 235–242.

[9] Jiqiang N., Dan Z., Feng L., & Yanping Y.. (2018). Effect of train length on fluctuating aerodynamic pressure wave in tunnels and method for determining the amplitude of pressure wave on trains. Tunnelling and Underground Space Technology, S0886779817305126-.

[10] Hui Yuan., Dan Zhou., & Shuang Meng. (2019). Study of the unsteady aerodynamic performance of an inter-city train passing through a station in a tunnel. Tunnelling and Underground Space Technology, 86, 1–9.

[11] Guang Z., Hyeon K. D., & Dong K. H.. (2018). Numerical studies on the radiation of train-tunnel impulse waves. Tunnelling and Underground Space Technology, 80, 211–221.

[12] Zhang G., Kim T. H., Kim D. H., & Kim H. D.. (2018). Prediction of micro-pressure waves generated at the exit of a model train tunnel. Journal of Wind Engineering and Industrial Aerodynamics, 183, 127–139.

[13] Uystepruyst D., William-Louis M., & François Monnoyer. (2013). 3d numerical design of tunnel hood. Tunn. Undergr. Space Technol. 38(9), 517–525.

[14] Zhang L., Liu H., Stoll N., & Thurow K., 2017(a) Influence of tunnel aerodynamic effects by slope of equal-transect ring oblique tunnel portal. J. Wind Eng. Ind. Aerod. 169, 106–116.

[15] Zhang L., Thurow K., Stoll N., & Liu H., 2018 Influence of the geometry of equal-transect oblique tunnel portal on compression wave and micro-pressure wave generated by high-speed trains entering tunnels. J. Wind Eng. Ind. Aerod. 178, 1–17.

[16] Niu J., Zhou D., Liang X., Liu T., & Liu S., 2017 Numerical study on the aerodynamic pressure of a metro train running between two adjacent platforms. Tunn. Undergr. Space Technol. 65, 187–199.

[17] Kim J. Y., & Kim K. Y., 2009 Effects of vent shaft location on the ventilation performance in a subway tunnel. J. Wind Eng. Ind. Aerod. 97(5–6), 174–179.

[18] Rabani M., & Faghih A. K., 2015 Numerical analysis of airflow around a passenger train entering the tunnel. Tunn. Undergr. Space Technol. 45, 203–213.

[19] Heine D., Ehrenfried K., Heine G., & Huntgeburth S., 2018 Experimental and theoretical study of the pressure wave generation in railway tunnels with vented tunnel portals. J. Wind Eng. Ind. Aerod. 176, 290–300.

[20] Xue P., You S., Chao J., & Ye T., 2014 Numerical investigation of unsteady airflow in subway influenced by piston effect based on dynamic mesh. Tunn. Undergr. Space Technol. 40, 174–181.

[21] López González, Marta, Galdo Vega, Mónica Fernández Oro, Jesús Manuel, & Blanco Marigorta, E., 2014 Numerical modeling of the piston effect in longitudinal ventilation systems for subway tunnels. Tunn. Undergr. Space Technol. 40, 22–37.

[22] Baker C. J., 1986 Train aerodynamic forces and moments from moving model experiments. J. Wind Eng. Ind. Aerod. 24(3), 227–251.

[23] Yang Q. S., Song J. H., & Yang G. W., 2016 A moving model rig with a scale ratio of 1/8 for high speed train aerodynamics. J. Wind Eng. Ind. Aerod. 152, 50–58.

[24] Kim J. Y., & Kim K. Y., 2007 Experimental and numerical analyses of train-induced unsteady tunnel flow in subway. Tunn. Undergr. Space Technol. 22(2), 166–172.

[25] Doi T., Ogawa T., Masubuchi T., & Kaku J., 2010 Development of an experimental facility for measuring pressure waves generated by high-speed trains. J. Wind Eng. Ind. Aerod. 98(1), 55–61.

[26] Baker C., Jordan S., Gilbert T., Quinn A., Sterling M., Johnson T., & Lane J., 2014 Transient aerodynamic pressures and forces on trackside and overhead structures due to passing trains. Part 1: Model-scale experiments; Part 2: Standards applications. Proceedings of the Institution of Mechanical Engineers, Part F: Journal of Rail and Rapid Transit, 228(1), 37–70.

[27] Sterling M., Baker C. J., Jordan S. C., & Johnson T., 2008 A study of the wakes of high-speed passenger trains and freight trains. Proceedings of the Institution of Mechanical Engineers, Part F: Journal of Rail and Rapid Transit, 222(2), 177–193.

[28] Soper D., Baker C., & Sterling M., 2014 Experimental investigation of the wake development around a container freight train using a moving model facility. J. Wind Eng. Ind. Aerod. 135, 105–117.

[29] Miyachi T., Fukuda T., & Saito S., 2014 Model experiment and analysis of pressure waves emitted from portals of a tunnel with a branch. J. Sound. Vib. 333(23), 6156–6169.

[30] Liu F., Yao S., Zhang J., & Zhang Y. B., 2016 Effect of increased linings on micro-pressure waves in a high-speed railway tunnel. Tunn. Undergr. Space Technol. 52, 62–70.

[31] Zhou D., Tian H. Q., Zhang J., & Yang M. Z., 2014 Pressure transients induced by a high-speed train passing through a station. J. Wind Eng. Ind. Aerod. 135, 1–9.

[32] Zhang L., Yang M. Z., Liang X. F., & Zhang J., 2017(b) Oblique tunnel portal effects on train and tunnel aerodynamics based on moving model tests. J. Wind Eng. Ind. Aerod. 167, 128–139.

[33] Gilbert T., Baker C. J., & Quinn A., 2013 Gusts caused by high-speed trains in confined spaces and tunnels. J. Wind Eng. Ind. Aerod. 121(5), 39–48.

[34] Niu J. Q., Zhou D., Liang X. F., Liu S., & Liu T. H., 2018 Numerical simulation of the Reynolds number effect on the aerodynamic pressure in tunnels. J. Wind Eng. Ind. Aerod. 173, 187–198.

[35] BS EN, 2006 Railway applications–aerodynamics-Part5: requirements and test procedures for aerodynamics in tunnels. BS EN 14067–5, 7–9.

[36] Zhang H., Zhu C., Zheng W., You S., Ye T., & Xue P.. (2016). Experimental and numerical investigation of braking energy on thermal environment of underground subway station in china\"s northern severe cold regions. Energy, 116, 880–893.

[37] Zhang H., Cui T., Liu M., Zheng W., Zhu C., & You S., et al (2017). Energy performance investigation of an innovative environmental control system in subway station. Building and Environment, 126, 68–81.

