# Supplementary material for: Moving model analysis on the transient pressure and slipstream caused by a metro train passing through a tunnel
Source: PLoS One. 2019 Sep 10;14(9):e0222151. doi: 10.1371/journal.pone.0222151 (PMC6736275; doi:10.1371/journal.pone.0222151)
Supplement: S1 Appendix — (DOCX) [file pone.0222151.s001.docx]

**S1 Appendix**

We studied the train model at a scale of 1/15 in order to verify the impact of the scaled value, as shown in Fig. 1. Comparing the 1/15 scaled model test results with the 1/10 test results, it can be seen from Fig. 2 that the pressure change laws of the two are basically the same. The pressure amplitude increases with a decrease in the Reynolds. According to Table 1, the maximum difference in pressure peak is 2.86%. This indicates that the scaled value (Reynolds number) has little effect on the train surface pressure.


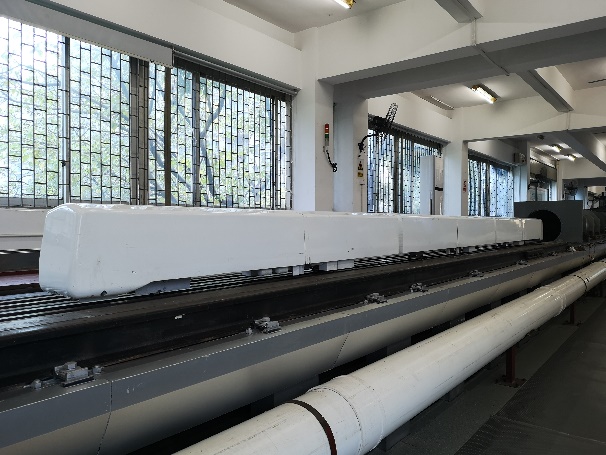

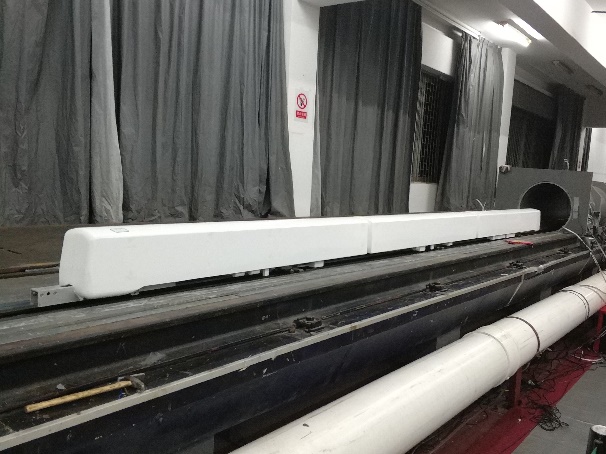


(a) 1/10-scale train model (b) 1/15-scale train model

**Fig. 1 train model**

**Fig. 2 Curves for the pressures**

**Table 1 ΔP of different scale trains**

|  | Measurement points (x/Ltr) | | | | | | | | |
| --- | --- | --- | --- | --- | --- | --- | --- | --- | --- |
|  | 0.05 | 0.16 | 0.27 | 0.38 | 0.5 | 0.61 | 0.72 | 0.84 | 0.95 |
| 1/10 | 2165 | 2105 | 2043 | 1767 | 1622 | 1415 | 1323 | 1204 | 1002 |
| 1/15 | 2198 | 2164 | 2087 | 1809 | 1657 | 1455 | 1359 | 1237 | 1029 |
| Diff./Ref.(%) | -1.54 | -2.79 | -2.14 | -2.39 | -2.15 | -2.86 | -2.70 | -2.75 | -2.69 |
